# Controllable Synthesis of Graphene by Plasma‐Enhanced Chemical Vapor Deposition and Its Related Applications

**DOI:** 10.1002/advs.201600003

**Published:** 2016-05-17

**Authors:** Menglin Li, Donghua Liu, Dacheng Wei, Xuefen Song, Dapeng Wei, Andrew Thye Shen Wee

**Affiliations:** ^1^ State Key Laboratory of Molecular Engineering of PolymersDepartment of Macromolecular ScienceFudan UniversityShanghai200433P. R. China; ^2^ Key Laboratory of Multi‐scale Manufacturing TechnologyChongqing Institute of Green and Intelligent TechnologyChinese Academy of SciencesChongqing400714P. R. China; ^3^ Physics DepartmentNational University of Singapore2 Science Drive 3Singapore117542Singapore

**Keywords:** controllable synthesis, graphene, growth mechanism, plasma‐enhanced chemical vapor deposition

## Abstract

Graphene and its derivatives hold a great promise for widespread applications such as field‐effect transistors, photovoltaic devices, supercapacitors, and sensors due to excellent properties as well as its atomically thin, transparent, and flexible structure. In order to realize the practical applications, graphene needs to be synthesized in a low‐cost, scalable, and controllable manner. Plasma‐enhanced chemical vapor deposition (PECVD) is a low‐temperature, controllable, and catalyst‐free synthesis method suitable for graphene growth and has recently received more attentions. This review summarizes recent advances in the PECVD growth of graphene on different substrates, discusses the growth mechanism and its related applications. Furthermore, the challenges and future development in this field are also discussed.

## 1 Introduction

Graphene, an atomically thin crystal with carbon atoms arranged into a honeycomb lattice, has attracted numerous interest due to its extremely high carrier mobility, ambipolar electric field effect, room temperature quantum Hall effect, low optical absorption, high specific area and thermal stability.^1^, ^2^, ^3^, ^4^, ^5^ Pristine graphene was originally obtained by mechanical exfoliation of graphite in 2004,^1^ versatile methods have since been developed toward synthesis of graphene, such as oxidation of graphite,^6^ liquid‐phase exfoliation,^7^, ^8^ chemical vapor deposition (CVD)^9^, ^10^, ^11^, ^12^ and thermal decomposition of SiC.^13^, ^14^ However, liquid‐phase exfoliated graphene usually suffers from structure defects and uncontrollable size, shape, layer numbers. Epitaxial growth of graphene on SiC substrates enables the synthesis of high‐quality wafer‐scale graphene,^15^ but the method is limited to high‐temperature (≈1500 °C), ultrahigh vacuum and expensive SiC substrates. CVD has been developed as an inexpensive approach for the synthesis of large‐area graphene with high quality on transition metal substrates. Till now, large‐area polycrystalline and millimeter‐sized single crystalline graphene domains have been achieved via CVD.^9^, ^10^, ^11^, ^12^, ^16^ However, for electronic applications, CVD graphene requires postgrowth transfer process from the surface of catalysts onto dielectric substrates, which induces contamination and structural defects, thus reduce the performance of graphene. Without a metal catalyst, graphene growth requires higher temperature. Several pioneering studies toward direct growth of graphene on dielectric substrates have been reported, which need higher temperature in the range from 1100 to 1650 °C.^17^, ^18^, ^19^

Plasma‐enhanced CVD (PECVD) has emerged as an important method for producing carbon materials such as diamonds, carbon nanotubes (CNTs), vertically oriented graphene (VG) nanosheets as well as graphene. The energetic electrons generated by the plasma boost the ionization, excitation and dissociation of hydrocarbon precursors at relatively low temperature. Thus, low‐temperature growth of graphene can be realized directly on desired substrates by PECVD in the absence of metal catalysts. Therefore, PECVD has attracted more and more attention as a promising method for controllable graphene synthesis. In this review, we summarize recent advances in plasma‐enhanced controllable synthesis of graphene including graphene and VG nanosheets. The growth mechanism and their potential applications such as flexible photovoltaic devices, field‐effect transistors (FETs), sensors, supercapacitors, and charge trapping memory (CTM) are also discussed in detail.

## 2 Brief Introduction to PECVD

**Figure 1:**
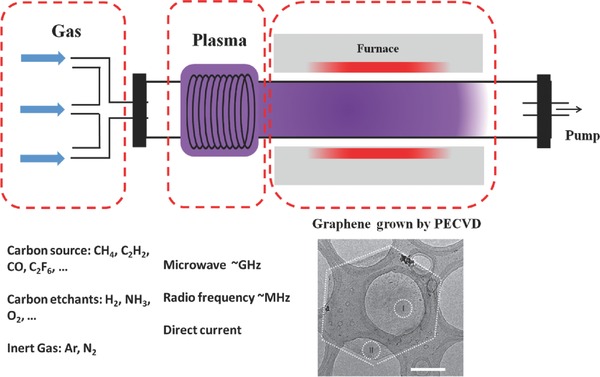
Schematic of experimental setup for PECVD including gaseous system, plasma generator system, and vacuum heating system.

The experimental setup for PECVD consists of three main parts including the gas, the plasma generator and the vacuum heating chamber, as illustrated in **Figure** **1**. Other setups where the plasma generators and the growth chamber are combined are also widely used in PECVD growth. Plasma generator is the core of the PECVD, can mainly be categorized into three types depending on the power source for plasma generation, i.e., microwave (MW) plasma (commonly 2.45 GHz), radio frequency (RF) plasma (commonly 13.56 MHz) and direct current (dc) plasma. The MW plasma is a type of high‐frequency electromagnetic radiation in the GHz range. To date, MW‐PECVD has been used extensively in the synthesis of graphene and its derivatives such as CNTs, nanowalls and diamond films. RF plasma is another popular source with domain frequency in MHz range. The energy of an RF generator is coupled to the plasma in three main modes: the evanescent electromagnetic (H) mode, the propagating wave (W) mode and the electrostatic (E) mode W‐mode. H‐mode inductively coupled plasma (ICP) has the advantage of high energy density and a larger plasma volume, thus yielding high growth rates. In contrast, E‐mode capacitively coupled plasma cannot be used as an independent plasma source due to relatively low energy. As the simple setup, dc glow plasma is also widely used source. There are two geometric designs for dc glow plasmas: parallel‐plate and pin‐to‐plate, which can produce uniform and nonuniform plasma sources, respectively.

Gaseous species are essential for the synthesis of graphene and its derivatives, which can be categorized into three functional groups. (i) The gaseous precursors containing carbon provides carbon radicals for graphene growth via plasma‐enhanced reaction. (ii) Gases such as H_2_, O_2_ are added as amorphous carbon etchants to produce high‐quality graphene and its derivatives. (iii) Gases such N_2_, NH_3_ normally are used to achieve the doping of as‐grown graphene, which can tailor the electrical properties of graphene.

During the plasma‐enhanced process, the source gas is activated by the energetic electrons generated in the plasma. The ionization, excitation, and dissociation of the source gases all occur in the low‐temperature plasma process. First, the ionization processes proceed via interactions between energetic electrons and gas molecules. Second, high‐energy ions generated in ionization processes subsequently react with source gas molecules. Finally, various radicals form via various dissociation reactions. These radicals are more reactive than ground‐state atoms or molecules, which enable the formation of graphene and its derivatives on catalyzed or noncatalyzed surfaces at low temperature. In order to optimize the synthesis process, the plasma‐enhanced process needs to be understood theoretically and experimentally.

**Figure 2:**
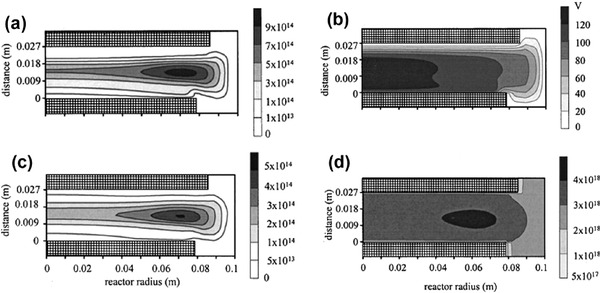
Calculated axial and radial species distributions of a) electrons; b) potential; c) ions; d) CH_3_ neutrals. Reproduced with permission.^24^ Copyright 2002, American Institute of Physics.

Simulations of the plasma process have been performed to optimize the growth parameters. A number of plasma models have been developed based on different gas systems.^20^, ^21^, ^22^ In these models, tens of species (ions, electrons, neutrals and radicals) and reactions are considered. For the methane or methane/hydrogen plasma, 8 neutrals, 11 ions and 5 radicals have been taken into account in 1D fluid model.^23^ 27 electron reactions, 7 ion‐neutral reactions and 12 neutral‐neutral reactions are included in this model. The densities of radicals and ions are found to vary with distance in the plasma based on 1D fluid models. Extending to the radial directions, 2D fluid model is proposed.^24^ According to the simulation results, the electron density reaches the maximum at the fringes of the electrodes where the potential *V* change dramatically, as shown in **Figure** **2**a,b. Thus, more frequent electron related reactions occur in these regions, leading to the formation of high‐energy ions and neutrals densities, as shown in Figure 2c,d.

**Figure 3:**
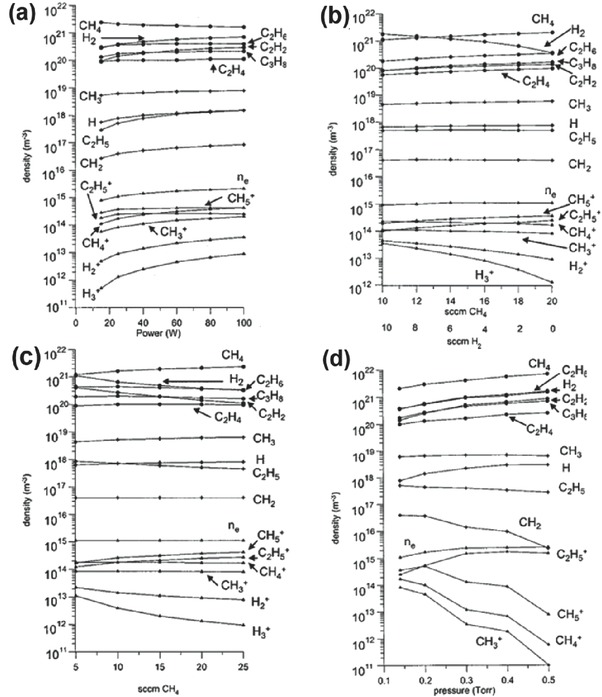
Calculated densities of nonradical neutrals, radicals, ions, and electrons as a function of a) radio frequency generator power, b) CH_4_ and H_2_ gas flow mixture, c) CH_4_, and d) pressure. Reproduced with permission.^23^ Copyright, 2001, American Institute of Physics.

The densities of ions and radicals are also influenced by the power, gas mixture ratio, gas flow and pressure. In the 1D fluid model of methane/hydrogen system, all species except methane increase slightly at higher plasma power due to more dramatical reactions among electrons, ions and radicals, as shown in **Figure** **3**a. It should be mentioned that effective power values (about 50% of the generator power) used in the model may be different from the actual power in the plasma. Hence, a good agreement cannot be obtained between the calculated and experimental results at low plasma power. The gas mixture ratio is also a key factor in species distributions. Nonradical neutrals (H_2_, CH_4_ C_2_H_6_, C_3_H_8_, C_2_H_4_, and C_2_H_2_) increase linearly with increasing CH_4_. Radicals (H, CH_2_, CH_3_, and C_2_H_5_) remain almost constant at different CH_4_/H_2_ ratios. For the ions, the H_2_^+^ and H_3_^+^ show a dramatic decrease with the rising of CH_4_ gas flow while slight increase could be observed for CH_5_^+^ and C_2_H_5_^+^ ions, as shown in Figure 3b. When increasing the total gas flow, less H_2_ and related ions (H_2_^+^ and H_3_^+^) form in the plasma, but other radicals and ions (CH_5_^+^, C_2_H_5_^+^, CH_4_^+^, CH_3_^+^) show no variations with increasing CH_4_, as shown in Figure 3c. The pressure is also believed to influence the reactions among electrons, ions, radicals and neutrals. In the fluid model, nonradical neutrals increase slightly as a function of pressure. Radical concentrations do not change with pressure. For ions, C_2_H_5_^+^ ions increase while the other ions (CH_5_^+^, CH_4_^+^, CH3^+^) drop drastically, as shown in Figure 3d.

Besides the CH_4_ and CH_4_/H_2_ systems, several models are also used for the Ar/CH_4_/H_2_,^25^ Ar/C_2_H_2_/H_2_,^26^ Ar/C_2_H_2_/NH_3_,^27^ CH_4_/NH_3_,^28^ and C_2_H_2_/NH_3_ 28, 29 systems. For gas systems including Ar, Ar atoms become the dominant neutrals in all species.^25^, ^26^ The densities of CH_4_ and H_2_ decrease more dramatically with the plasma power. For nonradical and radical neutrals, higher hydrocarbons (C*_x_*H*_y_*) are more likely to break into radical fragments due to more intense electron‐neutral reactions in Ar plasma, compared to the CH_4_ and CH_4_/H_2_ systems. In the plasma models containing NH_3_, additional 22 species, 43 electron‐impact reactions, 48 ion‐neutral reactions and 67 neutral‐neutral reactions should be considered. For the Ar/C_2_H_2_/NH_3_ systems,^27^ atomic hydrogen can be produced from C_2_H_2_ and NH_3_ dissociation reactions. It is speculated that hydrogen related species generated from the NH_3_ dissociation reactions produce evident etching effects on amorphous carbon structures which is important for the formation of pure CNT structures. Pure and above 30% C_2_H_2_ plasma results into amorphous carbon like films and obelisk‐like nanotubes.^29^

The plasma process is also investigated by experiments such as laser‐induced fluorescence spectroscopy (LIFS),^30^ infrared laser absorption spectroscopy (ILAS),^31^ mass spectrometry (MS),^25^, ^32^ and optical emission spectroscopy (OES).^25^ ILAS can be used to measure the densities of infrared radiated reactive species.^31^ The absorption lines of different species can be obtained for the concentration determination. By LAS, the densities of radicals (CH_3_, CH, and CH_2_) and neutrals (C_2_H_4_, C_2_H_6_) are found to change with plasma power. MS is a common method of monitoring species at the substrates, but it is difficult to assess unstable species.^32^ Hence, the MS analysis of species is limited to low C*_x_*H*_y_* neutrals and radicals. The OES provides the information of related ions in plasma processes.^25^ All the dominant ions, radicals and neutrals can be studied by MS and OES. Moreover, the reactions could also be analyzed by measuring the radicals, ions and neutrals.

Plasma‐enhanced process is a complicated process containing various kinds of species and reactions, which play an important roles in the plasma‐enhanced growth process of graphene. For example, the density of carbon related species can influence the morphology of the as‐grown graphene. Additional species such as H_2_, Ar can serve as amorphous carbon etchant toward high‐quality graphene. Other additional species such as NH_3_, N_2_ can dope graphene with heteroatoms and adjusting the electrical properties. All simulative and experimental results above offer information about these species and reactions, which contribute to understanding kinetic growth in the plasma process, which are of great significance to realization of the controllable synthesis of graphene.

## 3 Plasma‐Enhanced Growth of 2D Graphene

Graphene has been considered as an attractive candidate for future electronic materials due to its excellent electrical properties and atomic thickness. The 2D structure enables graphene to be adapted to current photolithography and integration processes. Among existing synthetic methods, CVD has been considered one of the most promising methods as it can grow high‐quality graphene films at relatively low cost. Large area polycrystalline and millimeter‐sized single crystalline graphene has been synthesized and applied in electronics.^9^, ^10^, ^11^, ^12^, ^16^ Compared to thermal CVD, PECVD possesses more potential in future electronic applications due to the advantages of low growth temperature and free posttransfer process. Until now, successful synthesis of high‐quality graphene via PECVD on metal, dielectric and 2D substrates has been reported.

### 3.1 Plasma‐Enhanced Growth of Graphene on Transition Metal Substrates

**Figure 4:**
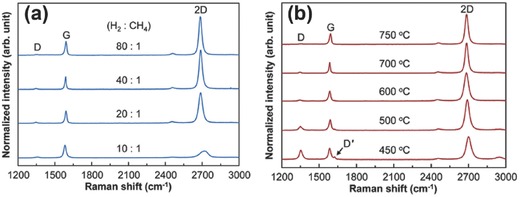
Raman spectra for graphene synthesized by microwave plasma‐enhanced CVD a) at various methane/hydrogen ratios, b) at different growth temperature. Reproduced with permission.^41^ Copyright, 2011, American Institute of Physics.

Thermal CVD growth of graphene on transition metal substrates usually requires a high temperature (800–1000°C), which is still too high for industrial production. Therefore, low‐temperature synthesis of graphene remains challenging for applications in electronics. PECVD can achieve the low‐temperature growth of high‐quality graphene films on transition metal substrates such as Ni,^33^, ^34^, ^35^ Cu,^36^, ^37^ Co,^35^, ^38^ and so on. Woo et al.^34^ synthesize uniform graphite films on Ni foils at low growth temperature of 850 °C by using remote RF‐PECVD. Pure ethylene is used as the carbon source. Raman spectra show negligible D peak, which indicate the high quality of as‐grown graphene films. Different ratios of G and 2D peak at different positions suggest that the thickness of graphene film is inhomogeneous from monolayer to multilayer. Similar high‐quality graphene films are synthesized at lower temperature ranging from 650 to 700 °C with a gas mixture of methane and hydrogen.^39^ The remote plasma configuration used in the work promotes the growth of planar graphene films with the electric field parallel to the substrates, rather than perpendicular electric field in other plasma configuration. Besides, large‐area surface wave plasma (SWP) is also used to produce large‐area graphene with growth temperature lower than 400 °C due to higher density plasma and radicals. By using the SWP–CVD, graphene‐like films is first grown on Al foil, although the melting point of Al is too low to be used as the substrate in CVD or PECVD.^40^ However, Graphene films fabricated by SWP–CVD on Cu and Al foils show a high density D peak and low density D' peak which are attributed to abundant edges and boundary effects. High‐quality monolayer graphene can be synthesized on the surface of transition metal by optimizing the growth parameters. Kim et al.^41^ demonstrate a low‐temperature synthesis of monolayer graphene on polycrystalline Ni foils by MW PECVD. In the work, monolayer graphene is obtained on polycrystalline Ni foils under various ratios of hydrogen and methane with growth temperature from 450 to 750 °C, as shown in **Figure** **4**a,b. The layer number of the as‐grown graphene depends on the gas mixture ratio. When the ratio of hydrogen and methane drop to 10:1, graphene with six layers rather than monolayer is grown on the Ni foils, as shown in Figure 4a. Although, the growth temperature can be decreased due to the high energy provided by MW plasma, more defects form during the growth process at low temperature. As shown in Figure 4b, higher D peaks can be observed with the growth temperature of 450 °C. An obvious D peak and shoulder peak (D′ peak) appear in the Raman spectrum. The D′ peak is mainly attributed to the low degree of crystalline at low growth temperature.

**Figure 5:**
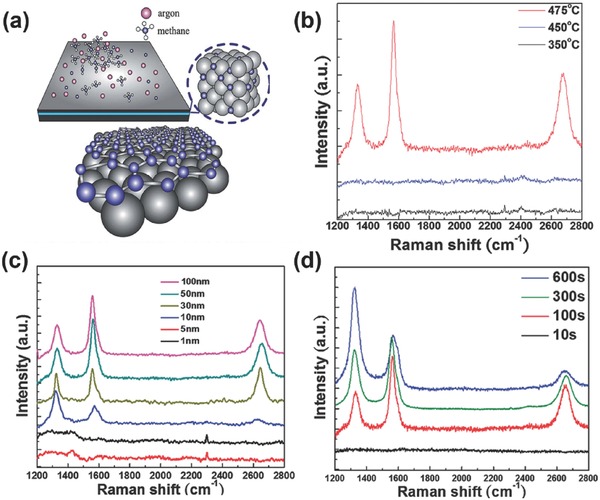
a) Carbon atoms dissolved in the interstices of nickel lattice and arranged into hexagonal ring structure on the surface of nickel during the precipitation. Raman spectra for graphene synthesized by radio frequency plasma‐enhanced CVD b) with different growth temperature, c) on Ni films with different thickness, d) with different deposition times. Reproduced with permission.^33^ Copyright 2013, Royal Society of Chemistry.

The typical dissolution precipitation occurs on transition metal with high carbon solubility such as Ni during the plasma‐enhanced growth process. The substrate temperature, the film thickness and the deposition time make obvious influence on the thickness and the crystalline structure of as‐grown graphene. Peng et al.^33^ have investigated the growth of graphene on Ni film by RF‐PECVD. The RF plasma is utilized to enhance the decomposition of methane into carbon species under hydrogen‐free conditions. The carbon species randomly dissolve in the interstices of Ni atoms. During the cooling process, the dissolved carbon species precipitate and arrange into hexagonal ring structure of graphene, as illustrated in **Figure** **5**a. The thresholds of growth temperature, film thickness and deposition time are investigated respectively. Below the threshold temperature of 475 °C, carbon species generated by RF plasma are unable to dissolve into the nickel films, thus no graphene films grow on the nickel films, as shown in Figure 5b. The quantity of dissolved carbon species depends on the nickel thickness. When the thickness of the nickel films is below 10 nm, the dissolved carbon species are not enough to form continuous graphene films on the surface of the nickel. No carbon related peaks could be observed in the Raman spectra. When increasing the thickness of nickel films to over 10 nm, amorphous carbon structures rather than graphene form without 2D peaks. When the thickness of nickel films is increased to over 30 nm, graphene layers with characteristic peaks (D, G, and 2D peaks) could be obtained, as shown in Figure 5c. Short growth time below 10 s fails to accumulate enough carbon species in Ni films to form graphene in the subsequent precipitaion process. Figure 5d shows that higher D peak and suppression of 2D peaks can be observed with longer growth time, indicating that not only sp^2^ but also sp^3^ carbon form in the growth process in the absence of hydrogen.

**Figure 6:**
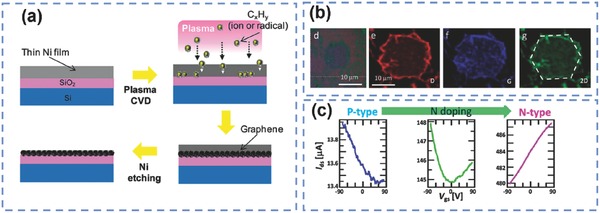
a) Schematic illustration of the direct growth of graphene on SiO_2_/Si with a ultrathin Ni film as the buffer layer: A nickel films is deposited on a SiO_2_/Si substrate; CxHy ions or radicals accelerated toward the nickel surface and diffuse into regions near the interface; carbon atoms preferentially precipate on the interface to form hexagonal graphene; graphene remians on the SiO_2_/Si substrate after chemically etching the nickel films. b) The optical and Raman maps of hexagonal graphene domains on the SiO_2_/Si grown by RH‐PCVD. c) The tranfer curve of the graphene grown by RH‐PCVD at 950 °C with NH_3_ flow rates of 0, 5, and 15 sccm, respectively. An obivious N doping can be observed from the left to the right. Reproduced with permission.^42^ Copyright 2012, American Chemical Society.

The dissolution precipitation occurs on not only the surface but also the underneath of transition metals. High‐quality monolayer graphene with a hexagonal domain has been synthesized along the interface between deposited Ni films and SiO_2_/Si substrate by the rapid‐heating plasma CVD (RH‐PCVD).^42^ The growth process is illustrated in **Figure** **6**a. During the plasma‐enhanced process, high‐energy carbon radicals generated in plasma are accelerated toward the substrate and penetrate through the surface of the deposited Ni films. As a result, the density of dissolved carbon inside nickel layer is higher than that at the surface, leading to selective growth at the interface between Ni and SiO_2_/Si substrate. At the initial growth stage, hexagonal domains of graphene with size of about 10–20 μm can be observed on the SiO_2_/Si substrate after etching the Ni layer, as shown Figure 6b. Similarly, large scale graphene films are also grown on the interface by adjusting the growth conditions. By using the Ni layer as the buffer layer, high‐quality graphene can be synthesized on the SiO_2_/Si substrate with high controllability in size and shape. Moreover, the electrical properties of the graphene can be tuned by introducing NH_3_ plasma during the RH‐CVD growth process. The negative shift in Dirac point can be seen in the transfer curve of N‐doping graphene grown under NH_3_ plasma, as shown in Figure 6c. Higher NH_3_ flow rates result into more negative Dirac point.

For metal substrate with low carbon solubility, the surface catalyzed dissociation mainly occurs during the graphene growth process. Polycrystalline Cu foil has been used as an excellent substrate for growth of high‐quality monolayer graphene over large area, due to its catalytic nature.^9^ Instead of dissolving and precipitating on the surface of the transition metals, the hydrocarbon precursors directly dissociate on the surface of Cu, then assemble into graphene structure. In PECVD system, the dissociation on Cu surface would be enhanced by plasma source. For the graphene growth on the surface of Cu, both the carbon radicals generated from surface catalysis and plasma excitation contribute to the graphene growth. For conventional CVD, the growth of successive layer graphene is dramatically slowed down due to loss of catalytic Cu surface after the first layer graphene forms. However, for PECVD, the reactive carbon radicals from plasma‐enhanced dissociation still contribute to the formation of successive layers at a relatively higher rate.^37^

For metal substrate with high carbon solubility and catalyzed surface, both dissolution precipitation and catalyzed dissociation occur during the growth process. For example, Co is a transition metal with a high carbon solubility of 4.1 at% and catalytic property for the dissociation of hydrocarbon precursors. It has been reported that few‐layer graphene can be successfully grown on polycrystalline Co films at 800 °C by RF‐PECVD.^38^ The first layer and the successive layers grow on the Co films by different mechanisms.^35^ The effect of deposition time on graphitic degree and in‐plane crystalline size has been investigated. Raman spectra show that all the graphene films deposited for different time (15, 40, 90, 360, 20 min) show similar characteristic peaks: D band, G band and 2D band. For short deposition time (15–40 s), the first layer graphene is formed by the enlargement of small graphene domains and in‐plane crystalline size increases during the growth process. When the growth time is increased to 90–360 s, the Co film has been covered with the first layer graphene, CH*_x_* radicals could not be dissociated into C_2_ by Co catalyzed dissociations, which are necessary for the 2D extension of graphene domains. CH*_x_* radicals generated in the plasma can terminate the growth of graphene domains and form new nucleation sites of graphene on Co film, leading to more edge and boundary structures on the Co film. Therefore, the ratio of I_D_/I_G_ in Raman spectra initially decreases and then increases with the growth process.

### 3.2 Plasma‐Enhanced Growth of Graphene on Dielectric Substrates

Large‐area graphene with high quality has been grown on transition metal substrates such as Ni or Cu foils by CVD. However, the metal‐catalyzed grown graphene requires posttransfer and catalyst removal for the applications in electronics. Chemical contamination and structural defects (wrinkles, or even breakage) cannot be avoided due to the metal etching process. It is widely believed that direct growth of graphene films on dielectric substrates would promote the widespread application of graphene films in future electronics. The pioneering study has reported synthesis of graphene on dielectric substrates by pre‐depositing sub‐micrometer‐thick Cu films as the buffer layer. The buffer Cu layer is then removed by evaporation in a high temperature low‐pressure atmosphere, but the evaporation of metal films still induces contaminations, wrinkles, and breakage in graphene films.^43^ Semiconductors and metal oxides have been reported as the catalysts for the growth of CNTs,^44^, ^45^, ^46^ indicating their possibility as substrates for the growth of graphene. Several reports have shown that graphene‐like films can be synthesized on Si, SiN, Al_2_O_3_, SiO_2_, and MgO.^47^, ^48^, ^49^ However, graphene‐like films grown by these methods normally suffer from poor crystalline quality and coexisting amorphous carbon. In order to improve the quality of the as‐grown graphene, higher growth temperature (1100–1650 °C) is necessary. Chen et al. have reported the direct growth of high‐quality polycrystalline graphene films on SiO_2_ and SiN substrates by thermal CVD.^18^, ^19^ The graphene films grown by this method exhibit excellent field effect mobility from 500 to 1000 cm^2^ V^−1^ s^−1^. However, the high growth temperature is not compatible with existing semiconductor technologies.

**Figure 7:**
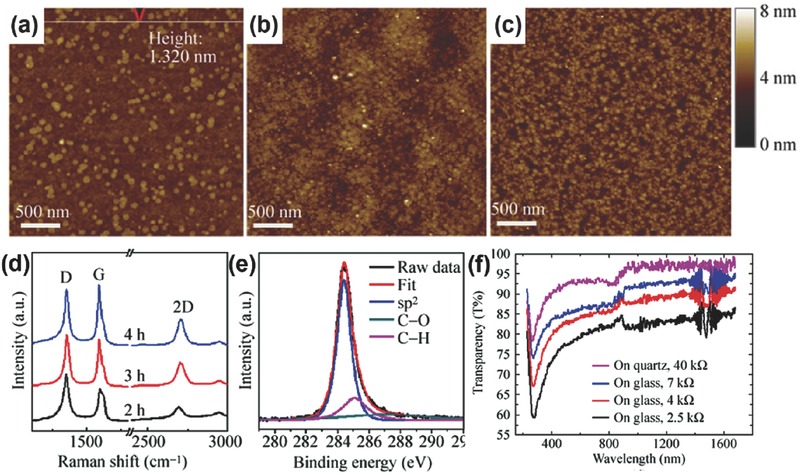
AFM images of nanographene grown on SiO_2_ with different growth time of a) 2 h, b) 3 h, and c) 4 h. An obvious nucleation and coalescence process observed during the growth. d) Raman spectra of the nanographene samples shown in panels (a), (b), and (c). All the samples featured with isolated D, G, and 2D peaks. e) XPS spectra of the as‐grown nanographene. The C 1s spectrum mainly fitted with sp^2^ carbon. f) Transmittance and corresponding resistance at different growth time. Reproduced with permission.^50^ Copyright 2011, Springer.

Plasma‐enhanced CVD has attracted much interest as an important method to achieve low‐temperature growth of graphene on dielectrics. Pioneering researches in this field demonstrate nanographene films growth on various substrates such as SiO_2_, atomic layer deposited (ALD) Al_2_O_3_, sapphire, quartz, mica, Si and so on at a relatively low temperature ≈550 °C.^50^, ^51^ The growth process of nanographene films on SiO_2_ substrates has been studied using atomic force microscope (AFM), as shown in **Figure** **7**a–c. In the early growth stage, nanographene islands uniformly nucleate on the SiO_2_ substrates with 1.2–1.5 nm thickness, corresponding to 2–3 layers graphene. With the continuous growth, higher density of nanographene islands form and coalesce into a continuous and uniform nanographene films. The as‐grown nanographene films consist of densely packed nanoislands. Raman spectra show characteristic peaks of a graphitic structure with D, G, and 2D peaks and the XPS C 1s spectrum is well fitted with a dominant peak assigned as sp^2^ carbon, as shown in Figure 7d,e. All these results indicate high crystalline nanographene films without amorphous carbon or diamond. The D peak is mainly due to small crystalline or edges of nanographene films. Nanographene films with various transmittance (85%–92%) and resistance (40–7 kΩ sq.^−1^) are obtained using different growth time, indicating that the optical and electrical properties of nanographene films could be adjusted by growth time, as shown in Figure 7f. It should be noticed that overall film resistance is mainly due to the resistance between nanogaphene clusters. Negative temperature‐dependence of the resistance can be observed due to the thermal generation of charge carriers.

With the advantage of direct growth on SiO_2_/Si substrates, two‐terminal nanographene field‐effect devices could be fabricated by electron‐beam lithography and lift‐off techniques without posttransfer process. The nanographene based devices exhibit an ambipolar field‐effect behavior with the mobility of 15 cm^2^ V^−1^ s^−1^. The mobility is far lower than graphene films grown by thermal CVD due to small crystalline size and numerous edges. Moreover, it should be mentioned that the gate modulation of nanographene based devices is very weak and the on/off ratio is close to 2. Similar nanographene films can be achieved on other substrates such as ALD Al_2_O_3_, sapphire, quartz, mica, Si, SiC.

**Figure 8:**
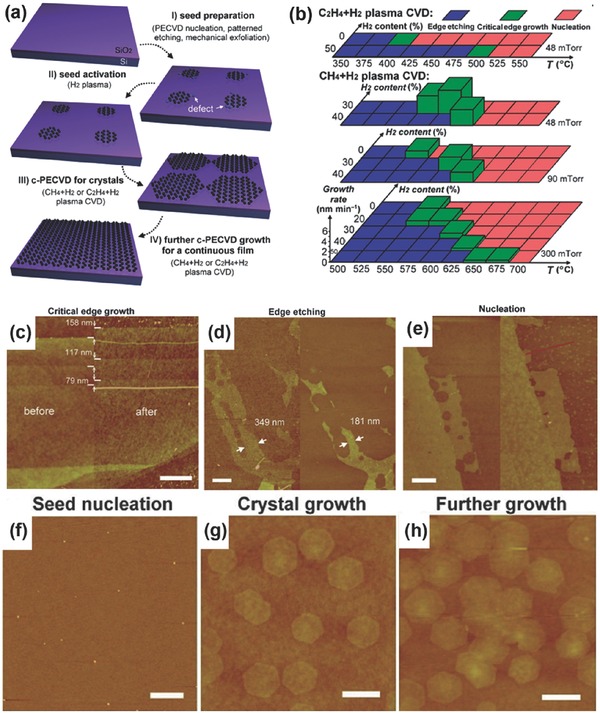
a) Schematic of PECVD growth process: seed preparation, seed activation, c‐PECVD growth for crystals, c‐PECVD growth for a continuous films. b) Illustrated regions for etching, nucleation and edge growth. Blue region: edge etching; green region: edge growth; red region: nucleation. AFM images of exfoliated graphene flake after PECVD c) critical edge growth; d) edge etching; e) nucleation. AFM images of the growth of HGCs on SiO_2_/Si by PECVD h) nucleation of graphitic clusters at 650 °C. HGCs on SiO_2_/Si after PECVD growth at 600 °C for g) 90 min and h) for 120 min. Reproduced with permission.^52^

The plasma‐enhanced CVD enables the growth of nanographene films on arbitrary dielectric substrates. However, applications of nanographene films in electronics are limited to weak gate modulation and low field effect mobility. In order to enhance the electrical properties of the as‐grown graphene, Wei et al. develop the critical crystal growth of graphene by introducing H_2_ plasma in PECVD.^52^ In the growth process, the catalyst‐free crystal growth of graphene is observed on substrates such as sapphire, HOPG, and SiO_2_/Si with the growth temperature as low as 450 °C. **Figure** **8**a illustrates PECVD growth process including seed preparation, seed activation, crystal growth and further growth into continuous film. All the exfoliated graphene flakes, nucleated graphene islands and patterned graphene could be used as further growth seeds. H_2_ plasma is introduced to activate edges for the critical crystal growth. Edge growth, edge etching and nucleation are observed during the plasma‐enhanced growth process, as shown in Figure 8c–e. The edge growth is observed at the edge of exfoliated graphene with three layer thickness on critical conditions. After 60 min plasma‐enhanced growth process, the edges of each layer move by 79, 117 and 158 nm respectively, as shown in Figure 8c. The edge etching occurs at lower temperature or higher H_2_ content with the width decreasing from 349 nm to 181 nm, as shown in Figure 8d. Opposite reaction conditions result into the nucleation of graphitic clusters with the thickness of monolayer graphene, as shown in Figure 8e. The critical conditions for growth, etching and nucleation have been systematically investigated, as shown in Figure 8b. Besides of exfoliated graphene, high‐quality hexagonal graphene crystals (HGCs) can be directly grown on HOPG or SiO_2_/Si substrates by using graphitic clusters as the grown seeds, as shown in Figure 8g–h. Small graphitic clusters are first nucleated at higher temperature (650 °C) and then grown into HGCs at critical growth temperature (600 °C). The calculated field effect mobility of the as‐grown HGCs are in the range of 550–1600 cm^2^ V^−1^ s^−1^, similar to the metal‐catalyzed CVD graphene and exfoliated graphene.

Although better electrical properties can be obtained with HCGs grown by critical PECVD, this method still suffers from low growth rate and high nucleation density. For example, the growth rate of the critical crystal growth by PECVD is normally below 10 nm min^−1^ (1 nm min^−1^ at 250 mTorr, 4.5 nm min^−1^ at 48 mTorr), which is far lower than the growth rate of metal‐catalyzed CVD. Moreover, the nucleation density of the PECVD can reach ≈10^7^ nuclei cm^−1^, which is about six orders of magnitude higher than CVD with reduced nucleation density (≈4 nuclei cm^−1^).^16^ Large domain single crystalline graphene has not been achieved by critical PECVD so far.

### 3.3 Plasma‐Enhanced Growth of Graphene on 2D Substrates

Although SiO_2_/Si substrates have been widely used as the common substrate for fabrication of the graphene devices, the electrical transport properties of graphene are largely limited to the surface roughness, impurities, charged surface states and large lattice mismatch of amorphous SiO_2_. 2D materials have been found to be better substrates due to its electrical properties and ultra‐flat surface. Graphene based devices exhibit high carrier mobility when using h‐BN as the substrate^53^ or encapsulated by molybdenum disulfide, tungsten disulfide or hexagonal boron nitride (h‐BN).^54^ As the insulting analogue of graphene, h‐BN has been explored as one of the best dielectric substrates for graphene‐based electronics, due to its atomically smooth surface,^55^ self‐cleaning and small lattice mismatch (1.7%) with graphite.^56^ The dielectric constant (ϵ ≈ 3–4) and breakdown voltage (*V* ≈ 0.7 V nm^−1^) of h‐BN is comparable to those of SiO_2_, which enables it an excellent gate dielectric material. The graphene‐based devices on h‐BN substrate show an attracting performance. Graphene/h‐BN hetero‐structures are fabricated via mechanical transfer process. For example, Dean et al. fabricated graphene devices on single crystal h‐BN substrates via poly‐methyl‐methacrylate (PMMA) based transfer techniques.^53^ The field effect motilities of graphene devices on h‐BN substrates are almost an order of magnitude higher than devices on SiO_2_/Si substrates. Moreover, the h‐BN can behave as an ideal tunnel barrier due to its large band gap (5.2–5.4 eV) and atomically thin structure like graphene. The alternative device fabricated by the h‐BN sandwiched by two graphene electrodes shows high on/off ratio of 10^6^, which provide another effective solution to low on/off ratio in graphene based devices.^57^ However, graphene/h‐BN devices fabricated by mechanical transfer normally suffer from unstable electrical performance due to chemical contamination, structure defects and uncertain alignment between graphene and h‐BN. Instead, graphene/h‐BN hetero‐structures are fabricated by CVD techniques.^58^, ^59^, ^60^, ^61^, ^62^, ^63^ The precise alignment between grown graphene and h‐BN provides more favorable device characters. For example, theoretical calculations predicts that AB stacked graphene/h‐BN could open a band gap of 53 meV in graphene.^56^ Recently, large single‐crystalline graphene domains up to 20 μm can be synthesized on h‐BN with the a gaseous catalyst silane.^63^ The Hall mobility can reach 20 000 cm^2^ V^−1^ s^−1^ and the secondary Dirac cone can be observed due to the moiré pattern.

**Figure 9:**
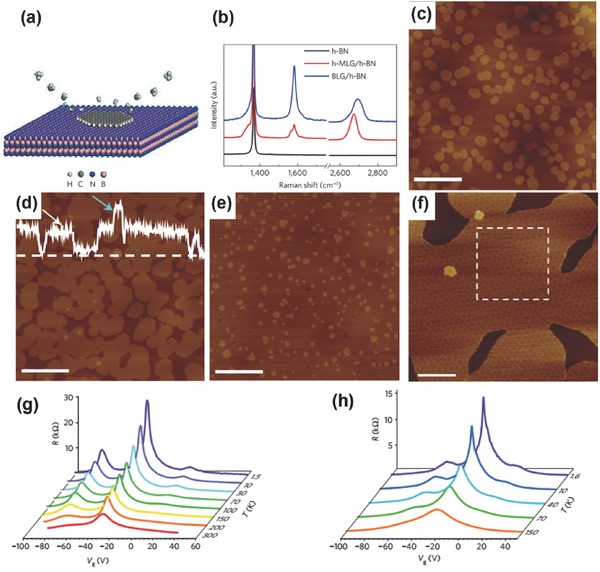
a) The illustration of the growth process on BN. b) Raman spectra for hexagonal monolayer graphene (h‐MLG) grains (red), bilayer graphene film (blue) and bare h‐BN surface (black). AFM images of graphene grown on h‐BN c) nucleated graphene domain, (d) enlarged graphene domain, (e) coalesced into a continuous film with second nuclei on top. The scale bars in panels (c)–(e) are 500 nm. f) Moiré patterns of as‐grown graphene domains on h‐BN. The scale bars in panel (f) is 100 nm. Resistance versus applied gate voltage measured at various temperature for g) monolayer graphene and h) bilayer graphene. Obvious resistance satellite peaks observed in monolayer graphene and lower satellite peaks observed in bilayer graphene. Reproduced with permission.^64^ Copyright 2013, Nature Publishing Group.

Direct growth of graphene on h‐BN by thermal CVD normally suffers from high growth temperature (above 1200 °C) and low growth rate. The PECVD is also used to achieve the low temperature (≈550 °C) epitaxial growth of graphene on h‐BN.^64^ The growth process is illustrated in **Figure** **9**a. The methane is dissociated into various reactive radicals for nucleation and growth of graphene at edges. Both monolayer and bilayer graphene have been grown on h‐BN with different growth duration. As shown in Figure 9b, the Raman spectra of mono‐ and bi‐ layer graphene grown on h‐BN are featured with the characteristic peaks of graphene and h‐BN. The splitted G peak in Raman spectra of monolayer graphene is observed indicating the zigzag edges of graphene domains. The 2D peak of bilayer graphene is fitted with four Lorentz curves, indicating a Bernal (AB) stacking. The epitaxial growth of graphene on h‐BN is similar to the growth of nanographene films. As shown in Figure 9c, small hexagonal grains of graphene nucleated on the h‐BN with the height of about 0.39 nm, indicating the formation of monolayer graphene domains. These small grains enlarge and coalesce for longer growth time, as shown in Figure 9d. Further growth result into the nucleation of the second layer graphene with the height of 0.77 nm, as shown in Figure 9e. All nucleated hexagonal graphene domains have the same orientations due to the Van der Waals epitaxial growth on h‐BN, which can coalesce into a continuous single crystalline graphene domain with enough growth time. Thus, higher carrier mobility can be expected in the epitaxial growth graphene films on h‐BN without the affecting of boundaries. Due to the mismatch between graphene and h‐BN, similar trigonal moiré pattern can be seen in the Figure 9f. All the moiré patterns are aligned with the zigzag edge. The trigonal moiré can lead to secondary Dirac cone in the as‐grown graphene, as shown in Figure 9g,h.

## 4 Plasma‐Enhanced Controllable Growth of VG Nanosheets

VG nanosheets are 3D internetworks of graphitic sheets arranged vertically on substrates. Because of the spatial alignment effect, VG nanosheets show some unique functional properties such as exposed reactive edges, high surface‐to‐volume ratio and high electrical conductivity. These properties enable VG nanosheets to have a wide range of applications as field emitters, supercapacitors, lithium‐ion batteries, gas sensors and biosensors. PECVD has used as a key synthesis technique for producing VG nanosheets.^65^, ^66^, ^67^, ^68^ for several years. Since surface‐bound vertically oriented carbon nanosheets were initially discovered by direct current (dc) arc discharge evaporation of graphite,^69^ PECVD with different plasma sources such as MW plasma,^70^, ^71^ dc plasma,^72^, ^73^, ^74^ RF plasma,^65^, ^75^, ^76^, ^77^ their combinations with different reactor configurations^78^, ^79^, ^80^ have also been used for the growth of VG nanosheets. The morphology and structure of VG nanosheers produced by PECVD are strongly dependent on the types of plasma sources^66^, ^67^, ^73^, ^79^, ^81^, ^82^, ^83^ and a series of operating parameters, including the feedstock gas type^84^, ^85^, ^86^, ^87^ (CH*_x_* (*x* = 1–3), CF_4_, CHF_3_, or C_2_F_6_), gas composition and proportion,^69^, ^86^, ^88^, ^89^ the substrate species^90^ and temperature,^75^, ^76^, ^80^, ^91^ the operating pressure^73^, ^76^ and the growth time,^75^, ^76^ and the plasma power.^67^, ^76^, ^92^

### 4.1 Plasma‐Enhanced Growth of VG Nanosheets with Controllable Morphology

**Figure 10:**
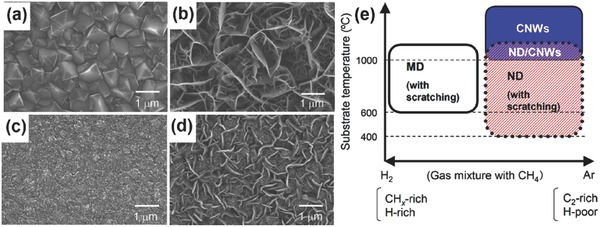
Top view of SEM images of a) microdiamond (MD), b) carbon nanowalls (CNWs), c) nanodiamond, d) ND/CNWs composite. e) The growth regimes for different morphologies: MD, ND, CNWs, and ND/CNWs composite with different growth temperature and gas compositions. Reproduced with permission.^93^ Copyright 2012, Institute of Electrical and Electronics Engineers.

VG nanosheets can grow into a variety of morphologies such as petal‐, turnstile‐, maze‐, and cauliflower‐like with different growth parameters. For example, different kinds of morphologies have been obtained by changing the feedstock gas type.^84^ Shiji et al. synthesized VG nanosheets with different morphologies by RF‐PECVD using CH_4_/H_2_, CF_4_/H_2_, CHF_3_/H_2_, and C_2_F_6_/H_2_ as the precursors respectively. The VG nanosheets could grow into not only thin and wavy in CH_4_/H_2_ system but also maze‐like morphologies in fluorocarbon/H_2_ systems. Moreover, the inter‐layer spacing of VG nanosheets increased with using CH_4_/H_2_, CF_4_/H_2_, CHF_3_/H_2_, and C_2_F_6_/H_2_ respectively. Teii et al.^85^ also obtained VG nanosheets with two kinds of morphology (pure VG and VG intercepted by diamonds) by MW‐PECVD using C_2_H_2_/N_2_/Ar and CH_4_/N_2_/Ar as the precursors respectively. It is found that the different morphology of VG nanosheets is due to the difference in the carbon dimer density. Low carbon dimer density of CH_4_/H_2_ system contributed to the formation of sp^3^‐C or amorphous carbon (a‐C) while high carbon dimer density promoted the growth of the pure VG networks. VG nanosheets with more ordered orientation and more uniform sheet height could be obtained in C_2_H_2_/H_2_ system, compared with CH_4_/H_2_ precursors.^93^ The growth regimes for microdiamond (MD), nanodiamond (ND), carbon nanowalls (CNWs) and ND/CNWs composite were proposed in **Figure** **10**e. It can be seen that higher growth temperature and rich carbon dimers facilitate VG nanosheet structures with less ND or microdiamond. The morphology of VG nanosheets grown under different conditions are also illustrated in Figure 10a–d. However, the morphology of VG nanosheets may be different under similar conditions using other plasma‐enhanced systems.

### 4.2 Plasma‐Enhanced Growth of VG Nanosheets with Controllable Density

**Figure 11:**
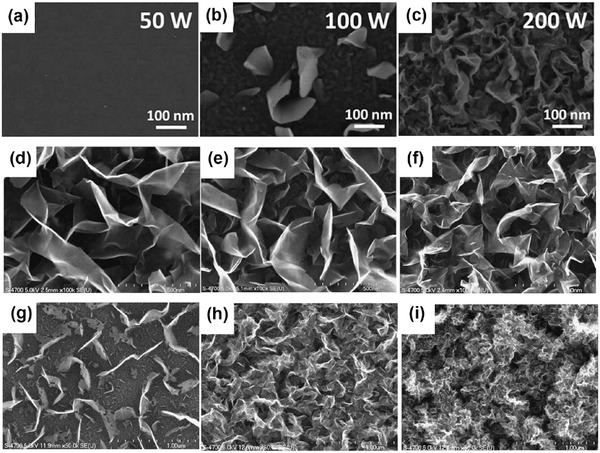
Top view of SEM images of VG nanosheets under different plasma power a) 50 W, b) 100 W, c) 200 W. Reproduced with permission.^92^ 2013, Royal Society of Chemistry. Top view of SEM images of VG nanosheets with different CH_4_ concentration d) 10%, e) 40%, f) 100%. Top view of SEM images of VG nanosheets at different growth temperatures: g) 630 °C, h) 730 °C, i) 830 °C. Reproduced with permission.^94^ Copyright 2004, Elsevier.

The density of VG nanosheets can vary depending on plasma power, gas composition, temperature and substrates. Yang et al.^92^ have found that the density of VG nanosheets is strongly depend on the plasma power during the growth process of RF‐CVD. Denser VG nanosheets can be obtained by increasing the plasma power from 50 to 200 W, as shown in **Figure** **11**a–c. The results indicate that the electric field aligned perpendicular to the surface provided by plasma play a more important role in the formation of nucleation centers for vertical growth. Wang et al.^94^ report controllable VG growth using CH_4_ diluted in H_2_ as gas precursors. The density of VG nanosheets is found to be strongly dependent on CH_4_ concentration and growth temperature. Higher nucleation density and smaller lateral size could be obtained with higher concentration CH_4_ (10–100%) and growth temperature (630–830 °C), as shown in Figure 11d,e and Figure 11f,g, respectively. However, growth temperature higher than 830 °C results into a high degree of corrugations. It is believed that the nucleation of VG nanosheets initiates at the boundary of buffer layer, which is a flat film comprising nanocrystalline graphene formed in the first growth stage. Different substrates induce different buffer layers. Davami et al.^90^ produced different densities of VG nanosheets on Si, Au/Si, Ni/Si and Cu substrates. Although VG nanosheets on these substrates have the similar leaf shape morphologies, VG nanosheets grown on Si substrate are denser and thinner than that on Si/Ni and Si/Au substrates.

### 4.3 Plasma‐Enhanced Growth of VG Nanosheets with Controllable Microstructure

All the carbon structures such as amorphous carbon, VG nanosheets, CNTs and diamond carbon can be produced by the PECVD. The effective removal of a‐C has been widely recognized as a crucial and inevitable step to the formation of high‐quality nuclei, and then further vertically grown VG nanosheets.^67^, ^79^ Several atoms and radicals play a key role in VG nanosheet growth. Zhu et al.^87^ reported the synthesis of VG nanosheets with CH_4_/H_2_ in an ICP system. Their work suggested that hydrogen atoms could act as effective etchant to remove the amorphous carbon. Shang et al.^95^ used plasma excited nitrogen species to remove the amorphous carbon in TM–MW system. Oxygen atom and hydroxy radicals were also reported to etch the amorphous carbon during the PECVD process and show stronger etchant ability than hydrogen radicals.^96^ The addition of Ar is able to provide high electrons and benefits the formation of radicals for VG growth. Goyett et al.^97^ found that the addition of Ar promoted the formation of C_2_ and H atoms, which benefit the growth of VG nanosheets. Based on OES measurement in a TM‐MW system, Teii et al.^85^ proposed C_2_ formed via direct dissociation reaction in C_2_H_2_/H_2_/Ar gas.

VG nanosheets usually cannot be obtained with pure methane as the precursor because of the absence of the etchant radicals. However, with the use of high‐intensity plasma source, VG nanosheets could be synthesized using pure methane as precursors.^94^, ^98^ Higher densities of H atoms and radicals can be obtained in these high energy plasma source systems, which act as effective amorphous C etchants.

## 5 Mechanism of Graphene Growth by PECVD

### 5.1 Nucleation and Coalescence Mechanism

In thermal CVD process, the graphene can be grown on the surface of the transition metals by surface catalytic decomposition mechanisms. The metal catalyzed growth of graphene involves surface processes including dissociation of hydrocarbon molecules, formation of C clusters, surface diffusion and extension of graphene nuclei.^99^ It is believed that the attachment of C clusters generated by surface catalytic decomposition to graphene nuclei is very important for the growth of high‐quality graphene films in metal catalyzed CVD. However, the nucleation process is largely enhanced due to more reactive radicals generated in plasma‐enhanced dissociation reactions. The nucleation and coalescence would be more important for plasma‐enhanced growth of the graphene, especially at low temperature or without metal catalysts.

**Figure 12:**
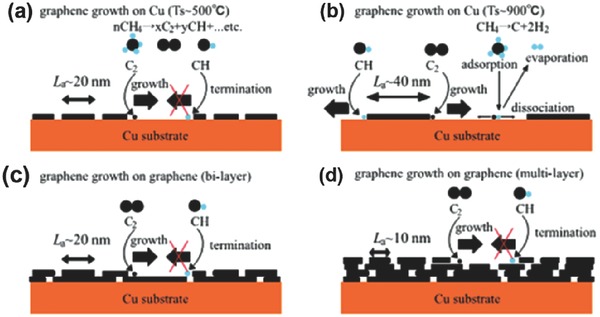
Schematic of growth mechanism on Cu substrate by PECVD. a) Monolayer growth at low growth temperature (500 °C). b) Monolayer growth with larger grain size at high growth temperature (900 °C). c) Secondary layer growth on the first layer. d) Successive layers growth. Reproduced with permission.^37^ Copyright 2012, Elsevier.

Koichiro Saiki et al. have reported the growth of graphene by PECVD on catalytic metal surfaces, and related mechanisms are also discussed in detail.^37^ It has been widely reported that Cu surface has a catalytic effect that dissociates hydrocarbons into the activated carbon species. However, the catalyzed dissociation process cannot occur at the temperature below 600 °C. At low growth temperature, only activated carbon radicals generated by the plasma contribute to growth process of graphene on Cu surface. The graphene growth process is dominated by nucleation and coalescence of graphene patches with size of ≈20 nm, as shown in **Figure** **12**a. Increasing the growth temperature to 900 °C, the catalytic effect of Cu arises. In the graphene growth process, carbon radicals can be generated by plasma‐enhanced and metal‐catalyzed dissociation. The activated carbon radicals generated by catalytic dissociation can enlarge the size of nucleated graphene patches to ≈40 nm, as shown in Figure 12b. After the growth of the first layer graphene on Cu, activated carbon radicals cannot be generated by metal catalyzed dissociation. Small graphene patches rather than enlarged graphene domains nucleate and coalesce into the second layer graphene, as shown in Figure 12c,d. Successive layer graphene can also be grown by the mechanism of nucleation and coalescence with smaller crystal size ≈10 nm. The growth of the graphene on noncatalyzed substrates can be fully dominated by nucleation and coalescence mechanism. As reported by Zhang et al. catalyst‐free growth nanographene films can be achieved on noncatalyzed SiO_2_/Si, Al_2_O_3_, mica, silica and even glass with low growth temperature.^50^, ^51^ Nanographene islands with different sizes and heights first form in the growth process. With longer growth duration, more nanographene islands nucleate and coalesce into continuous films.

### 5.2 Etching and Growth Mechanism

**Figure 13:**
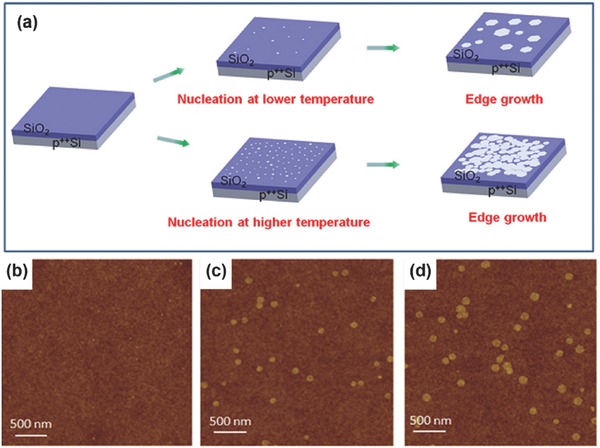
a) Different two‐step growth strategies for nanographene growth including isolated stages: nucleation and edge growth. The AFM images of a) nucleation at 560 °C, b) edge growth at 536 °C for 2 h, d) further edge growth at 510 °C for 2 h. Reproduced with permission.^100^ Copyright 2014, Elsevier.

In the nucleation and coalescence mechanism, nanographene with domain sizes varying from few to tens of nanometers nucleate on the substrates. The nanographene should be enlarged by edge growth rather nucleation in order to obtain high‐quality graphene films. Liu et al. present the competition of etching and growth mechanism in the catalyst‐free growth of the graphene by PECVD.^100^ In the mechanism, etching and growth processes are found to be dependent on the growth temperature with inverse trend. According to the mechanism, a two‐step strategy is proposed, in which nucleation and edge growth occur in two isolated stages, as shown in **Figure** **13**a. Nucleation at lower temperature followed by edge growth results into larger graphene domains that nucleation at higher temperature. It is expected that continuous polycrystalline graphene film with larger size graphene domain could be obtained via edge growth after low density nucleation process, as shown in Figure 13b–d.

**Figure 14:**
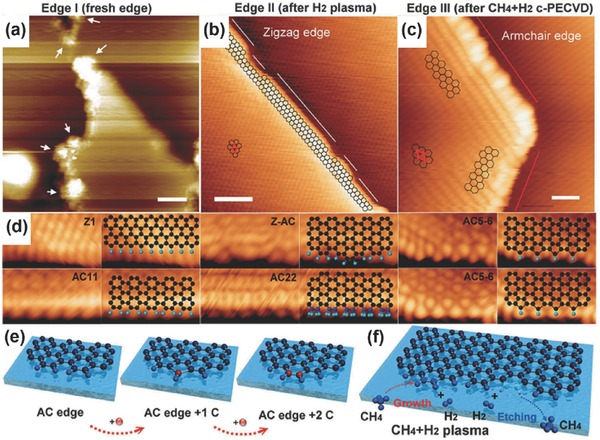
STM images of edges a) mechanical exfoliation; b) H_2_ plasma etching; c) CH_4_/H_2_ plasma CVD. d) STM images of several typical edge configurations and the corresponding atomic structures. e) Schematic illustration of edge growth by the sequential addition of two carbon atoms (red) to an armchair (AC) edge (C, blue; H, cyan). f) Schematic illustration of the reversible equilibrium in c‐PECVD. Reproduced with permission.^52^

Similar competition of etching and edge growth is also reported by Wei et al. in critical crystal growth of graphene.^52^ H_2_ plasma is known to etch the graphene from edges.^101^ After moderate H_2_ plasma treatment, macro‐structural defects are removed and atomically smooth hexagonal configurations form at the edges, as shown in **Figure** **14**a,b. The final edges show a preferential zigzag orientation after the H_2_ plasma treatment according to the previous first‐principles calculations.^102^ The zigzag oriented smooth edges in moderate H_2_ plasma can serve as the active sites for the crystal growth of graphene in the CH_4_/H_2_ plasma. After growth, the zigzag configurations transform into armchair configurations, which is in agreement with previous theoretical studies,^102^, ^103^ as shown in Figure 14c. In order to reveal the critical crystal growth mechanism in atomic scale, scan tunneling microscopy (STM) studies are performed to characterize the edge structures. Several typical edge configurations including zigzag (Z1), armchair (AC11, AC22), and zigzag‐armchair (Z‐AC) can be observed for edge and edge , according to the stimulated STM patterns, as shown in Figure 14d. Moreover, pentagon‐hexagon armchair edges (AC5‐6) can be occasionally observed on newly grown edge, because π electron on pentagon edge atoms can be identified from electrons on hexagon edge atoms in STM images. It is believed that AC5‐6 should be the transition state in growth process, as shown in Figure 14e. The competition between H_2_ plasma etching and CH_4_ plasma growth in c‐PECVD is illustrated in Figure 13f.

### 5.3 Vertical Growth Mechanism

**Figure 15:**
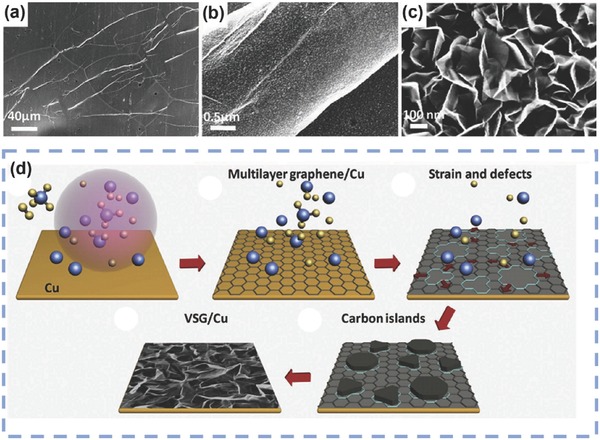
a) SEM image of planar multilayer graphene films grown on copper foil. b) Magnified SEM image of panel (a). c) SEM image of VSG films with growth time increasing to 2 min. d) Schematic illustration of the growth process of VSG films on copper foil in an MP‐CVD system. Reproduced with permission.^106^

Wu et al. first report the successful synthesis of CNWs on sapphire substrate.^104^ It is believed that the change of direction of electric field contributes to the formation of CNWs rather than CNTs. It is also reported that horizontally aligned CNTs can be grown on modified SiO_2_/Si, which can direct the electric field from plasma to the substrate surface.^105^ Therefore, the direction of electric field is essential for the growth of VG nanosheets. However, the growth mechanism of VG nanosheet remains unambiguous. Jiang et al.^106^ report that wafer‐sized and uniform vertically standing graphene (VSG) films on Cu foil can be grown by using MP‐CVD system.^106^ In order to reveal the growth mechanism, the evolution of VSG films is monitored by varying the growth time. A multilayer graphene films with wrinkles or ripples can be observed with growth time of 1 min, as shown in **Figure** **15**a,b. As the growth time increase to 2 min, the VG nanosheets can be observed in Figure 15c. This phenomenon suggests that VG film take place after 2D growth. Figure 15d shows the mechanism of the growth of the VSG films. First, the hydrocarbon is decomposed and absorb on the surface of the Cu, leading to the growth of 2D multilayer graphene films. Then the layer growth turns into vertical growth due to strain and defects accumulated in the as‐deposited films. Moreover, MW plasma can ensure the 3D growth of vertical graphene nanosheets, because reactive carbon radicals generated in MW plasma would reach the edge frequently and thus diffuse outward. Although the VG nanosheets have been widely grown by PECVD on various substrates, the vertical growth mechanism of VG nanosheets needs further investigations. The electric field should be considered for the growth direction and the transition from 2D growth to vertical growth should be investigated in detail.

## 6 Applications of Graphene Grown by PECVD

### 6.1 Application in Photovoltaic Devices

Graphene has potential applications in future photovoltaic devices, due to its high transparency (98%) and extremely low sheet resistance (≈25 Ω sq.^−1^). Till now, graphene films and graphene based composites have been exploited as transparent flexible electrodes in dye‐sensitized solar cells^107^, ^108^, ^109^ and organic photovoltaic devices.^110^, ^111^, ^112^ The graphene materials are normally transferred to desired substrates after CVD growth on the surface of transition metals. The contamination, wrinkles and breakage cannot be avoided during the mechanical transfer process, which limit the applications as the transparent electrodes in photovoltaic devices. It has been reported that nanographene films can be synthesized on arbitrary substrates at low temperature by PECVD with high transmittance (85%–92%) and relatively low sheet resistance (40–7 kΩ sq.^−1^).^50^, ^51^ Further investigations on the applications as transparent electrodes are required. Moreover, the sheet resistance of nanographene films remain higher than CVD grown graphene on transition metals due to abundant edges and small crystalline size. Reducing the density of nucleation by reasonable synthetic strategies is required for low sheet resistance.

Carbon based heterostructure junction solar cells have been reported in many previous studies.^113^, ^114^, ^115^, ^116^, ^117^, ^118^ Various hetero‐structures (Schottky and p–n junction) have been successfully fabricated based on amorphous carbon/n‐Si,^113^ CNTs/n‐Si^114^, ^115^ and graphene/n‐Si.^116^, ^117^, ^118^ The graphene based Schottky junction is more favorable due to its large built‐in field (0.55–0.75 V) and high charge separation efficiency. However, there remain many challenges in improving the power conversion efficiency (PCE) of graphene based Schottky junction solar cells. The use of high‐quality graphene and reasonable device fabrication can improve the performance of devices. Graphene/Si Schottky junction solar cell was fabricated by directly growing graphene‐graphitic films on Si substrate by PECVD.^118^ However, the PCE of graphene/Si Schottky junction solar cell was found to be 0.078% due to the poor quality of graphene‐graphitic films. Introducing effective etchant can remove defective structures from as‐grown graphene‐graphitic films, which may contribute to improving the performance of the graphene based Schottky junction solar cell.

**Figure 16:**
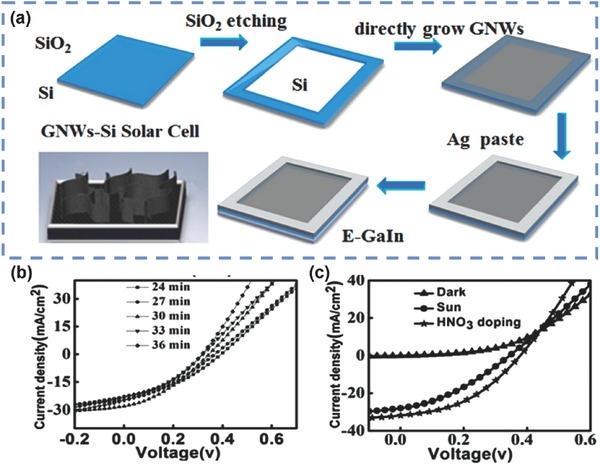
a) The fabrication process of GNWs/Si Schottky junction solar cells. b) *J*_sc_–*V*_oc_ curves of GNWs/Si Schottky junction solar cells with different growth times: 24, 27, 30, 33, and 36 min. c) Dark and light *J*_sc_–*V*_oc_ curves of GNWs/Si Schottky junction solar cells before and after HNO_3_ doping. Reproduced with permission.^123^ Copyright 2015, American Institute of Physics.

Graphene nanowalls (GNWs) are networks of graphene sheets and can also be used in carbon‐based solar cells.^119^, ^120^, ^121^, ^122^, ^123^ PECVD grown and plasma post treated GNWs have been used as a counter electrode in dye‐sensitized solar cell (DSSC).^120^ The DSSC with as‐deposited and H_2_ plasma treated GNWs showed an PCE of 1.64 % and 2.23% respectively. The reason for increase of energy conversion efficiency is the reduced sheet resistance due to H_2_ plasma treatment. The GNWs/Si heterojunction solar cells have similar device structures with graphene based Schottky junction solar cells, as shown in **Figure** **16**a.^123^ By directly growing GNWs on Si substrate via PECVD, GNWs/Si hetero‐junction solar cell could be fabricated with PCE of 3.1% and the energy conversion efficiency was increased up to 5.1% after chemical modification. The PCE of the GNWs/Si hetero‐junction solar cell can be enhanced by extending the growth time or p‐type doping, as shown in Figure 16b,c. As for GNWs based electrodes, its vertical orientations and wall‐like structures provide large surface area and reactive sites, which makes GNWs excellent electrodes. However, the high sheet resistance is one of the main challenges in the applications of GNWs as electrodes.

### 6.2 Application in FETs

Graphene is a 2D material with ultrahigh mobility and ambipolar field effect. The room temperature field effect mobility of graphene based transistors have been proved to be as high as 15 000 cm^2^ V^−1^ s^−1^, indicating promising applications in digital logic devices and high frequency devices.^1^, ^124^ Although mechanical exfoliated graphene has the highest quality, it cannot be used in integrated device fabrication. PECVD can achieve the controllable synthesis of the large‐area graphene on dielectric substrate. Recently, graphene has been grown on various substrates containing metals SiO_2_/Si sapp, hire, mica, h‐BN for applications in FETs.^34^, ^39^, ^50^, ^52^, ^64^, ^125^ PECVD grown graphene films on transition metals have similar growth process (dissolution precipitation and surface catalyzed dissociation) with thermal CVD grown graphene films, thus have comparable field effect motilities. Although the growth temperature of PECVD is lower than thermal CVD, the graphene films still need to be transferred from transition metals to dielectric substrate for applications in FETs. Nanographene films can be directly grown on dielectric substrates by PECVD. Two‐terminal nanographene field‐effect devices can be directly fabricated without post transfer process. However, small crystalline size or edge defects formed in nucleation and coalescence process result in field effect motilities as low as 15 cm^2^ V^−1^ s^−1^. Moreover, nanographene field‐effect devices show weak gate modulation and low on/off ratio. More reasonable synthetic methods are required for graphene films with less boundaries and edges.

**Figure 17:**
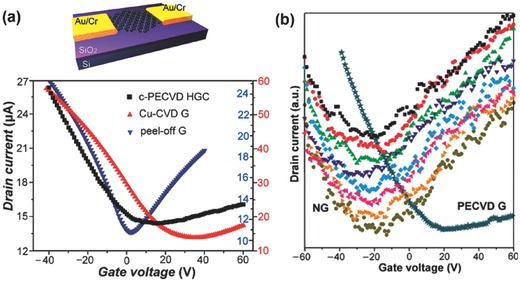
a) Transfer characteristics of FETs based on H_2_/CH_4_ plasma‐enhanced grown graphene (black), Cu‐CVD graphene (red), and peel‐off graphene (blue). Reproduced with permission.^52^ 2013, Wiley‐VCH. b) Transfer characteristics of eight FETs based on CH_4_/NH_3_ plasma‐enhanced grown NG and an FET based on H_2_/CH_4_ plasma‐enhanced grown pristine graphene. Reproduced with permission.^125^ Copyright 2015, American Chemical Society.

Catalyst‐free crystal growth of graphene on SiO_2_/Si was observed in the C_2_H_4_/H_2_ plasma.^52^ HGCs can be grown on SiO_2_/Si with the size of about 1 μm. FETs based on HGCs show high mobility in the range of 550–1600 cm^2^ V^−1^ s^−1^, as shown in **Figure** **17**a. It can be seen that the FETs of PECVD grown graphene have comparable mobility values to Cu‐CVD graphene and peel‐off graphene. For the applications in FETs, the electrical characteristic graphene should be modulated to be p‐type or n‐type. The amorphous nitrogen doped carbon film has been obtained with NH_3_/CH_4_ mixtures by PECVD.^126^ However, the amorphous nitrogen doped carbon film normally suffers from poor electrical transport and gate modulation due to its disordered structure. Recently, the crystal growth of nitrogen‐doped graphene (NG) has been achieved in NH_3_/CH_4_ plasma.^125^ The FETs of NG are fabricated using the growth substrate as the dielectric and gate electrode. An obvious negative shift of Dirac point could be observed in the transfer curve of NG FETs, indicating typical n‐type semiconductor behaviors, as shown in Figure 17b. The field effect mobility of NG is in the range of 100–400 cm^2^ V^−1^ s^−1^, higher than amorphous nitrogen doped carbon film (10 cm^2^ V^−1^ s^−1^) and comparable to NG (200–450 cm^2^ V^−1^ s^−1^) grown on transition metal. The defect‐free crystalline structure formed the etching and growth process contributes to the outstanding electrical properties of NG films, which shows potential in the future FETs.

However, the mobility of HGCs and NG grown by PECVD is still far lower than that of single crystalline graphene.^16^ Further improvement in the quality of HGCs and NG is also required. The higher mobility can be achieved by using better substrate without charged traps instead of amorphous SiO_2_/Si substrates. h‐BN is an excellent substrate with atomically smooth surface and small lattice mismatch with graphene. The similar 2D structure and Van der Waals between h‐BN and graphene favor the epitaxy growth in PECVD process. The mobility of graphene films grown on h‐BN by PECVD show the higher mobility of about 5000 cm^2^ V^−1^ s^−1^, compared with graphene grown on SiO_2_/Si substrates.^64^ Therefore, dielectric substrates is critical for the further improvement in the quality of graphene grown by PECVD.

### 6.3 Application in Supercapacitors

Supercapacitors (electric double‐layer capacitors and pseudocapacitors) have attracted much interest as a new energy storage device because of excellent charge/discharge rates, long cycle life, and high power density.^127^, ^128^, ^129^, ^130^ Electric double layer capacitors (EDLCs) operate based on rapid separation and adsorption of ions on the surface of the active materials. Porous carbon materials with high specific surface area such as activated carbon (AC), mesoporous carbon, and CNTs have been widely used as active materials in supercapacitors.^131^, ^132^, ^133^ GNWs is developed as an alternative supercapacitors' active materials in view of its high surface area, high conductivity and low contact resistance Its unique vertical structure facilitate the diffusion of ions.^82^, ^134^, ^135^, ^136^ Compared with other porous materials (ACs and graphene stacks), GNWs‐based supercapacitors show excellent capacitive behaviors even at relatively high frequencies. For example, a high frequency (120 Hz) alternating current (ac) line‐filtering can be realized with CNWs‐based supercapacitors due to ultrafast dynamic response.^137^ Later on, EDLC which can operate at kilohertz alternating current is also reported using VG nanosheets grown on nickel foam collectors.^138^

**Figure 18:**
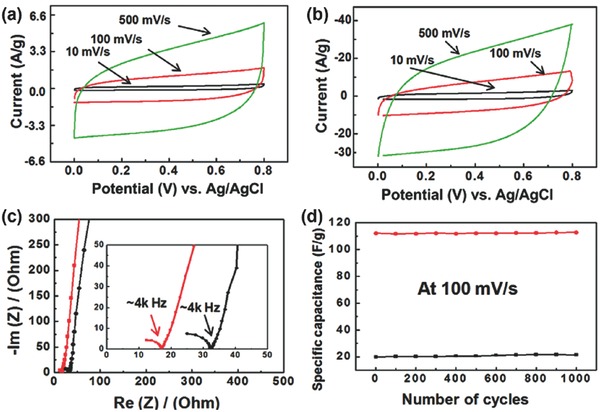
Cyclic voltammetry curves of VGNSs grown at a) 40% H_2_ and b) 80% H_2_. c) The impedance spectra of VGNSs grown at 40% H_2_ (black) and 80% H_2_ (red). d) The cycle stability of VGNSs grown at 40% H_2_ (black) and 80% H_2_ (red). Reproduced with permission.^136^

Recently, vertical graphene nanosheets (VGNSs) was directly synthesized on Ni foams with natural precursor butter at low temperature.^136^ The as‐grown VGNSs adhered to the Ni foams without using nonconductive polymeric binder. The supercapacitors based on VGNSs exhibit high specific capacitance 230 F g^−1^ at a scan rate of 10 mV s^−1^ and negligible capacitance after 1500 cycles at high current density, as shown in **Figure** **18**a,b. Furthermore, the morphology and structure of VGNSs can be adjusted by modifying the plasma power, gas precursor and growth parameters, which leading to various capacitive behaviors.^75^, ^136^ It can be suggested that thinner edge planes and higher graphitization contribute to a lower charge transfer resistance and better specific capacitance,^136^ as shown in Figure 18c,d. The specific capacitors can be improved by the combination of 1D CNTs and 2D VGs.^135^ In contrast to EDLCs, pseudocapacitors work by reversible Faradaic‐type redox reactions of ions. Although the pseudocapacitors usually have a higher specific capacitance, devices suffer from a lower power density and poor cycling stability compared with EDLCs. High‐performance pseudocapacitors can be realized by combining the VG and metal oxides or electrically conducting polymer. For example, pseudocapacitors with hybrids MnO_2_‐VG showed similar electrochemical behaviors as EDLCs. Similar applications of VGs decorated with MnO_2_ of diverse morphologies and other transition metal oxides have also been reported.^139^, ^140^

### 6.4 Application in Sensors

**Figure 19:**
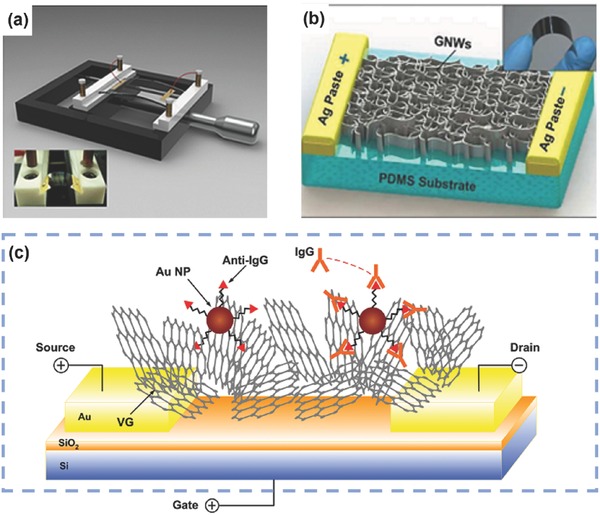
Schematic of a) graphene based strain sensor. The process of applying strain to the devices is shown in the picture. Reproduced with permission.^142^ 2012, American Institute of Physics. b) Flexible GNWs/PDMS temperature sensor. Two‐terminal device is fabricated by brushing two Ag paste on sides of CNWs. Reproduced with permission.^144^ 2015, Royal Society of Chemistry. c) VG biosensors. Anti‐IgG is anchored to VG nanosheets surface through Au nanoparticles. Reproduced with permission.^145^ Copyright 2013, Natue Publishing Group.

Recently, graphene based materials have been widely studied for sensing applications, e.g., in strain sensors,^52^, ^141^, ^142^, ^143^ temperature sensors,^144^ biosensors,^145^, ^146^, ^147^, ^148^ and gas sensors,^72^, ^81^, ^149^ as shown in **Figure** **19**. Nanographene films grown by PECVD have been transferred onto polydimethylsilxane (PDMS) with prestrain.^51^ It is found that the resistance of rippled graphene on PDMS linearly increases with applied strain. The strain sensors based on nanographene films could sustain a high tensile strain over 30% due to its high flexibility. Another advantage of nanographene based strain sensor was that gauge factor could be varied in the range from 10 to 10^3^ by adjusting the growth temperature because higher temperature resulted in higher nucleation sites, leading to a higher gauge factor.^143^

Wearable temperature sensors have been widely used in applications, such as electronic skins, robot sensors and human‐machine interface. CNWs transferred onto PDMS substrates are found to be three orders higher temperature coefficient of resistivity than other graphene materials due to excellent stretch ability of GNWs and large expansion large of PDMS.^144^ Thus, wearable temperature sensor could be fabricated by combination of CNWs and PDMS, as shown in Figure 19(b). The device has precise temperature measurement with intervals of 0.1 °C from 35 to 36 °C indicating that it could be used in monitoring the human body temperature.

The carbon nanomaterials normally achieve high sensitivity in the detection of bimolecular due to its extremely sensitive surface. The biosensor has been fabricated by direct growth of VG sheets on the sensor electrode through PECVD.^145^ After deposition of Au NP‐antibody conjugates on the VG surface, the device exhibits a significant change in the electrical conductivity when binding with target protein. Compared with drop‐casting method, the biosensor shows higher stability and repeatability with selective detection to specific protein. The one‐step method to prepare VG nanosheets based biosensors holds huge potential in scalable fabrications.

When absorbing gas molecules (CO, NO_2_, H_2_O, or NH_3_) on the surface, graphene based devices also show increase or decrease in conductivity. GNWs fabricated on metal electrodes by dc plasma‐enhanced CVD can respond to relatively low concentrations of NO_2_ and NH_3_, suggesting a low cost effective method to fabricate large‐scale gas sensors.^72^

### 6.5 Application in Charge Trapping Memory

CTM is nonvolatile flash memory based on the insulating charge storage layer. Due to the low‐dimension, chemical stability, and high‐work function, graphene is considered to be a potential candidate material for memory application.^2^, ^150^

**Figure 20:**
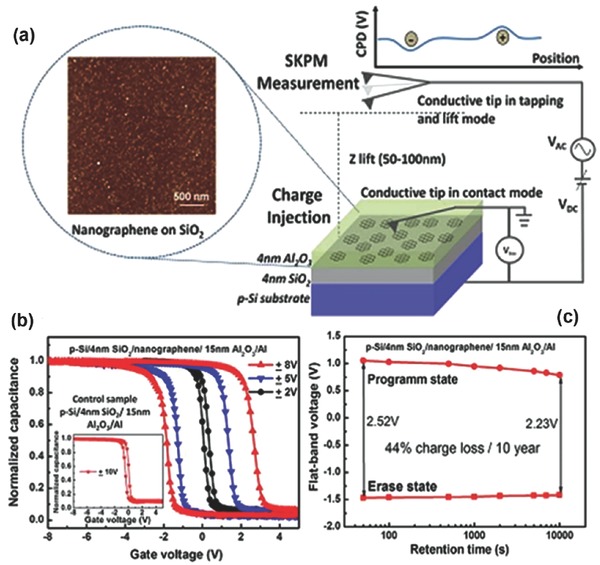
a) Schematic diagram of the SKPM measurement process for the Al_2_O_3_/nanographene/SiO_2_ structure. Inset: a typical AFM image of the as‐grown nanographene. b) High‐frequency CV characteristics under different gate voltage sweepings. c) Data retention for CTM. Reproduced with permission.^151^ Copyright 2013, Nature Publishing Group.

Due to the abundant edges, chemical and thermal stability, low cost, and capability with complementary metal oxide semiconductor (CMOS) devices, nanographene is considerate to be a good candidate as charge trapping material. Zhang et al. have reported nanographene based CTM.^151^ This novel nanographene charge‐trapping layer CTM includes the following layered structures: heavily doped substrate (p‐Si substrate), tunneling layer (4 nm SiO_2_), charge storage layer (nanographene), blocking layer (4 nm Al_2_O_3_) and gate electrode, as shown in **Figure** **20**a. The feasibility of nanographene as trapping material is investigated using the scanning Kelvin probe microscopy (SKPM) to test the surface potential variation. The results show that nanographene growth by PECVD has highly controllable charge trapping capacity with large trapping density, ultrathin thickness, and well uniformity.

The capacitance–voltage (CV) characteristics under different sweep voltages are shown in Figure 20b. From the data, CV curves reveal different memory window under different dual‐direction gate voltage sweeping. When the voltage sweep from −8 V to +8 V, a large memory window of 4.52 V can be obtained. The large memory window proves that the nanographene have the effect on the charge storage. Contrast data without nanographene shows no memory window which further proves the charge trapping effect of nanographene. Figure 20c depicts the data retention characteristics at room temperature. A 2.52 V memory window shrinks to 2.23 V after 10^4^ s. A charge loss of 44% after 10 years' operation is also predicted from data which may be caused by tunneling of neighbor nanographene.

## 7 Summary and Outlook

In summary, we discuss controllable synthesis of graphene and its derivatives by PECVD and its related applications. Both 2D graphene and VG nanosheets have been synthesized by PECVD on various substrates. Compared with thermal CVD, the growth temperature could be adjusted to be compatible with the level of the Si‐based electronics with the aid of the plasma. More importantly, the successful growth of graphene materials on dielectric, conducting and semiconducting substrates promotes the applications in FETs, sensors, energy conversion and storage devices. However, it remains challenging to realize practical applications of PECVD grown graphene. (i) wafer‐scale synthesis of high‐quality graphene on dielectric substrates would promote its applications in electronics, but PECVD growth rate is too low for industrial‐scale production. For example, the growth rate of critical crystal growth of graphene is below 10 nm min^−1^. (ii) The growth of single crystalline graphene contributes to higher motilities and more stable electronic properties. Recently, millimeter‐sized single crystalline graphene domains have been achieved on transition metals by conventional CVD, but large domain size of single crystalline graphene has not been synthesized on dielectric substrates by PECVD. (iii) The performance of graphene based devices can be further improved by using more favorable 2D materials. For example, h‐BN is a superior insulting substrate for graphene based device. The graphene grown on h‐BN exhibits higher mobilities with small band gap. Moreover, atomic thin h‐BN also can be used as the tunnel barrier in vertical graphene device. Besides h‐BN, other 2D materials should be explored as the substrates for graphene growth. (iv) VG nanosheets, as a 3D graphene network, have been widely used in various applications, such as photovoltaic devices, supercapacitors and sensors. The controllable synthesis of VG nanosheets with different morphologies and structures benefits the improvement in device performance. In the future, systematic studies are required toward better controllability. In conclusion, PECVD is a more promising method for controllable synthesis of graphene and its derivatives, and should be further explored.
